# Meetings of Societies

**Published:** 1915-12

**Authors:** 


### MEETINGS OF SOCIETIES.

### Bristol Medico-Chirurgical Society.

*Annual Meeting, October 13th,* 1915.

After a vote of thanks to the retiring President, Dr. Newnham, had been passed, Dr. George Parker took the chair, and gave an introductory address (see p. 129). Later a cordial vote of thanks was given to Dr. Parker for his able and interesting address.

The Acting Honorary Secretary (Financial), Dr. J. A. Nixon, read the annual report and presented the balance sheet.

The Honorary Librarian, Mr. C. K. Rudge, then read the following report :—

The medical wing of the Blind Asylum having been pulled down preparatory to the building of a New Library, etc., it has again been necessary to find other quarters for the Medical

Library. The Library is now in a suite of five rooms in the old Drill Hall, the entrance to which is from University Road. These rooms are admirably adapted for library purposes, being very well lighted and heated. The Library and Reading Room are lofty and lighted from above, and there is plenty of floor space. It was regrettable to have to report that owing to the unsatisfactory financial state of the Society it has not been possible to purchase any new books during the past year.

The Library of the Bristol Medico-Chirurgical Society now contains 12,961 volumes. During the year 94 volumes had been added. Of this number 75 were sent for review in the Society's *Journal,* and the remainder had come by gift or exchange. The Library is in regular receipt of 163 periodicals, all exchanges. Since last year the purchase of 28 serial publications had been discontinued for lack of funds.

The Bristol Medical Library, to which members have access, now contains 23,702 volumes, and 225 current British and Foreign periodicals. The number of volumes in the various libraries is as follows :—

BOOKS.

| | |
|---|---|
| Medico-Chirurgical Society .. .. | 12,961 |
| University Medical Library .. .. | 8,978 |
| Bristol Royal Infirmary .. .. | 1,189 |
| Bristol General Hospital .. .. | 574 |
| Total .. .. | 23,702 |

CURRENT PERIODICALS.

| | |
|---|---|
| Medico-Chirurgical Society .. .. | 163 |
| University Medical Library .. .. | 62 |
| Total .. .. | 225 |

The following are the elected officers of the Society for the Session 1915–16 : President—G. Parker, M.A., M.D. Cantab. ; President-Elect—F. H. Edgeworth, M.A., M.D. Cantab., D.Sc. Lond. ; Ex-President — W. H. Newnham, M.D. ; Editor of *Journal*—P. Watson-Williams, M.D. ; Hon. Librarian—C. King Rudge ; Editorial Secretary—J. M. Fortescue-Brickdale, M.D. ; Hon. Secretary—A. J. Wright, F.R.C.S. ; Committee—R. Shingleton-Smith, M.D., G. Munro Smith, M.D., I. Walker Hall, M.D., J. Lacy Firth, M.S., F.R.C.S., J. O. Symes, M.D., L. E. V. Every-Clayton, M.D., F.R.C.S., R. G. P. Lansdown.

---

*December 8th,* 1915.

Dr. George Parker, President, in the Chair.

Capt. Carey Coombs read a paper on **Heart Strain** as seen in Military Hospitals. The President and Drs. Michell Clarke and Watson-Williams discussed the paper. (See p. 149.)

Lieut.-Col. Michell Clarke read a paper on **Gun-Shot Injuries of Nerves.** The President and Messrs. Moore and Lansdown discussed the paper.

Major Lansdown read a paper on **The Localisation of Bullets.** (See p. 157.)

Capt. Stack read a paper on **Gun-Shot Wounds of the Eye** (See p. 198.)

### Obituary.

